# Happiness connects: The impact of mood on self-other integration

**DOI:** 10.3389/fpsyg.2022.986965

**Published:** 2022-11-17

**Authors:** Jing Zhang, Bernhard Hommel

**Affiliations:** ^1^Institute of Psychological Health, Hangzhou Dianzi University, Hangzhou, China; ^2^Faculty of Psychology, Technische Universität Dresden, Dresden, Germany

**Keywords:** self-representation, plasticity, affective states, metacontrol, self-other integration

## Abstract

Converging evidence suggests a considerable plasticity of self-representation and self-other boundaries. But what are the factors controlling this plasticity? Here we explored how changes in an individual’s affective state impact his/her self-other representation. Participants watched short videos to elicit happiness or sadness before rating unfamiliar faces with happy or sad expressions. After watching the happy video, participants showed more self-other integration of happy than sad faces, while watching the sad video reduced integration for both happy and sad faces equally. This finding suggests the interaction of two processes: Positive mood biases metacontrol toward flexibility, which fosters the processing of features in which self and other might overlap, and possible overlap increases self-other integration. Negative mood, in turn, biases metacontrol toward persistence, which focuses processing on strictly task-relevant feature dimensions, so that possible overlap is less likely to have an impact.

## Introduction

When we reach for a cup of water, we do not need to search for our hands first—we in some sense know who we are. This kind of “self-evidence” raises a fundamental philosophical and psychological question: how do we feel and know our bodies? The answer to this lies in how people represent and recognize themselves. The relationship between my body and “I” is very different from the relationship between my body and others, and the individual’s ability to recognize that s/he is different from the outside marks the formation of self-awareness. Human infants can only pass the mirror self-recognition task after a certain age, and Gallup et al. (2003) argue that self-recognition is a measure of self-awareness and a basis for making inferences about the mental states of others.

Self-recognition presupposes a distinction between the self and others, which, in turn, is based on how individuals represent these two. In other words, self-representation is the basis of self-recognition and the perceived ownership over one’s body (Gallagher, 2000). The rubber hand illusion (Botvinick and Cohen, 1998) and its variants (such as the virtual-hand illusion: e.g., Zhang et al., 2015; Zhang and Hommel, 2016) provide substantial evidence that body ownership is more variable and plastic than one may think. In fact, a few minutes of simultaneous visual-tactile stimulation (e.g., visual stroking of the object and tactile stroking of one’s real hand) can make participants feel ownership over an external object without any connection to one’s body. More recent developments of the basic design have extended these observations to the human face (Tajadura-Jiménez et al., 2012) and the entire human body (Ehrsson, 2007; Van Der Hoort et al., 2011). These studies imply that the integration of information from simultaneous multisensory channels may influence the outcome of self-representation (Tsakiris, 2017). In other words, the distinction between self and others is plastic, which might have come in favor of human evolution (Meltzoff, 2005), for self-other overlap seems to mediate how we understand and empathize with others (Cooke et al., 2018) and even benefit positive responses to mimicry (Hale and Hamilton, 2016).

Observations that the self-other distinction is not fixed but variable raise the question when and how this distinction changes and which principles these changes may underlie. There is a consensus that perceiving the states of others facilitates the activation of personal, affective, and conceptual representation (Preston and Hofelich, 2012). The present study focused on a possible role of emotions and affective states in determining the degree of self-other distinction. Previous theorizing suggests that affective states might impact self-other distinction through two mechanisms. First, recent extensions of the Theory of Event Coding (TEC; Hommel et al., 2001) to self- and other- representation (Hommel, 2018; Hommel, 2019; Hommel, 2021; Quintard et al., 2021) predict that self-other distinction is negatively predicted by feature overlap between self and other. That is, other things being equal, the more features self and other share the more the representation of oneself should consider the other and integrate the other into one’s self-concept—provided that the dimensions on which these features are defined are currently task-relevant or otherwise salient (Memelink and Hommel, 2013). Besides, evidence from social cognitive neuroscience supports the existence of a shared representation network between self and others, which underlies the identification and discrimination of self and others (Decety and Sommerville, 2003; Gallese, 2003). With respect to affective states, this implies that sharing emotions and affective states (e.g., sharing the feature of being happy) with someone else should reduce the distinction between myself and this other and increase self-other integration.

The second mechanism that might render emotions and affective states relevant for self-other integration is metacontrol. This mechanism refers to the fact that adaptive behavior requires a continuous balance between cognitive persistence and flexibility (Goschke and Bolte, 2014). According to the Metacontrol State Model (Hommel, 2015; Hommel and Colzato, 2017), metacontrol biases toward persistence render information processing highly focused, selective, competitive, exclusive, and serial, whereas biases toward flexibility are associated with inclusive, integrative, and parallel processing. Applied to self- and other- integration, this implies that a bias toward persistence would increase self-other distinction (due to strong mutual competition between the representations of self and other), whereas a bias toward flexibility would promote self-other integration (Hommel, 2018). Given that positive-going affective states have been associated with metacontrol biases toward flexibility (Dreisbach and Goschke, 2004; Akbari Chermahini and Hommel, 2012; Hommel and Colzato, 2017), one would thus expect that inducing positive mood should increase self-other integration.

The present study sought to test these two predictions by combining two experimental factors. For one, we attempted to induce positive or negative mood by presenting participants with what we considered happy or sad videos, and confirmed the successful induction by means of affect ratings. Second, we presented participants with unfamiliar faces showing happy or sad facial expressions and asked them to rate the closeness between themselves and this face by means of the Inclusion of Other in the Self (IOS) Scale. Since its development, the IOS scale has been used effectively in various studies regarding relationships and can assess self-other closeness as good as or even better than other more complex and lengthy measures (Aron et al., 2004). Our first prediction was that, based on the feature-overlap principle, the degree of self-other integration (as indicated by the IOS score) should depend on the match between the participant’s own current mood state, as induced by the video, and the expression of the face. More specifically, integration should be higher if the fit between own state and shown expression is high (happy video/happy expression, sad video/sad expression) than if it is low (happy video/sad expression, sad video/happy expression). Our second prediction was that, based on the metacontrol theory, self-other inclusion should in general be more pronounced after the induction of positive, as compared to negative mood.

## Materials and methods

### Participants

Eighty student volunteers (40 males and 40 females, mean age = 22.79 years, *SD* = 2.197, range 19–28) from Hangzhou Dianzi University, unfamiliar with psychological experiments, participated in exchange for course credit or pay. All participants were physically healthy and had no history of mental health problems. Ethical approval for this study was obtained from the local Psychology Research Ethics Committee, and written informed consent was obtained from all participants.

### Design and materials

We used a 2-factorial within-participants design. The two factors were video type (happy vs. sad video) and facial expression (happy vs. sad facial expression). The experiment consisted of two blocks. Each participant watched the happy video and the sad video sequentially (the content was balanced across participants, i.e., participants who watched the happy video in the first half of the experiment would watch the sad video in the second half, and vice versa). After each viewing, they would see a happy face and a sad face and needed to rate the overlap between themselves and the pictures (the computer randomly assigned the order of the happy and sad expressions).

We used two different videos as emotion priming materials, as indicated by a meta-analysis showing that film scenes are the most effective way of priming mood (Westermann et al., 1996). First, we downloaded 20 videos of 30 s duration from a popular short-video platform (TikTok) that were rated as happy or sad by users. Students who had not watched these videos were invited to rate the level of “happy” and “sad,” with “1” being the lowest and “10” being the highest. Twenty-eight people participated in rating the happy videos, and thirty-two people participated in rating the sad videos. Based on the rating results, one video with the highest level of happiness (*M* = 7.78, *SD* = 2.006) and one with the highest level of sadness (*M* = 7.88, *SD* = 2.211) were selected as the emotional primes for this experiment.

Facial expression pictures were made by composing multiple photos of real people and processing them with face morphing software, so to disguise their identity. We first invited volunteers to the lab for the photoshoot, and the models were asked to maintain as neutral a facial expression as possible. We took photos of 16 male and 16 female faces with neutral expressions in this process. The photos were then synthesized in groups of 8 photos of the same gender by the synthesis software (FaceFusion) to obtain four synthesized photos of the faces (there were two different pictures for each gender). To process the synthesized faces with neutral expressions, we recruited thirty volunteers and asked them to complete the following task: moving the degree bar to get an expression that they thought naturally represented happiness or sadness with the same software. We then averaged the results and used them as a criterion to generate the corresponding happy/sad expressions.

### Measurements

#### Affect grid

We adopted the Affect Grid (AG; Russell et al., 1989) to assess the effect of the video on participants’ affective states. The AG is a single-item scale that has been widely used in situations that require rapid and repeated assessment of an individual’s subjective affective state (Ma et al., 2016). This scale consists of a 9 × 9 grid, where the horizontal axis indicates the level of pleasantness, ranging from unpleasant feelings (“1**”**) to pleasant feelings (“9”), and the vertical axis indicates the level of arousal, ranging from sleepiness (“1”) to high arousal (“9”). Participants were required to assess their emotional state after watching the video twice during the experiment. To avoid the interference of numerical or alphabetic information on their choices, we presented them with a blank grid with explanations on horizontal and vertical coordinates.

#### Including other in the self scale

The Including other in the self (IOS) scale is a pictorial measure of the relationship between self and other, in which “self” and “other” are symbolized by two circles, respectively. Participants are asked to select the arrangement of the two circles that best describes their relationship between themselves and the specific “other” (Aron et al., 1992; Paladino et al., 2010). These arrangements differ with respect to the overlap between the two circles, which vary from distant and non-overlapping (level 1) to complete overlap (level 7; e.g., Ma et al., 2016). The metaphor of overlapping circles is considered a simple but successful way to represent (the relationship between) self and others, for it might correspond to how people process self-other related information in relationships (Aron et al., 2004). We presented all seven arrangements in a row, ranging from non-overlap to complete overlap, and asked the participants to use a computer mouse to click the most appropriate arrangement.

#### Experimental setup

The experiment was performed on a computer, and all data (age, gender, video types, AG scores, and IOS scores before and after viewing the different facial expressions) were recorded automatically in the database. Participants sat in front of the computer screen and performed the appropriate actions with the mouse. Before the experiment started, they had enough time to read the instructions and decide whether to continue or withdraw from the experiment. After confirming the start of the experiment, the participants selected their gender to ensure that they would see a face of the same gender as their own. After a “+” lasting for 500 ms, a composite photo with a neutral facial expression and the same gender as the participants appeared on the computer screen. Below the photo, there was the IOS scale, and the participants needed to select the most appropriate option according to their current perceived self-other overlap. Afterward, the participants watched a happy or sad short video of 30 s duration. They were allowed to control the starting time. After the video, the AG would be presented on the screen. Participants were instructed to click on the corresponding position in the grid according to their current affective state. Then, the same face but with different facial expressions appeared again with the requirement to judge the overlap degree. The photo was presented in animation, starting with a neutral face for 2 s (the same as the face the participants had seen before), and then the neutral expression gradually changed to a happy facial expression or a sad expression within 5 s. Once the animation was over, the IOS scale appeared again, and the participants could select the corresponding number to indicate their felt overlap degree with the new expression. This process was repeated twice, separated by a “+” lasting for 500 ms, with the expressions reversed, and random parameters of the program controlled the order of presentation. Specifically, after watching the video, the participants either saw the happy expression, then the sad expression, or vice versa.

**Figure 1:**
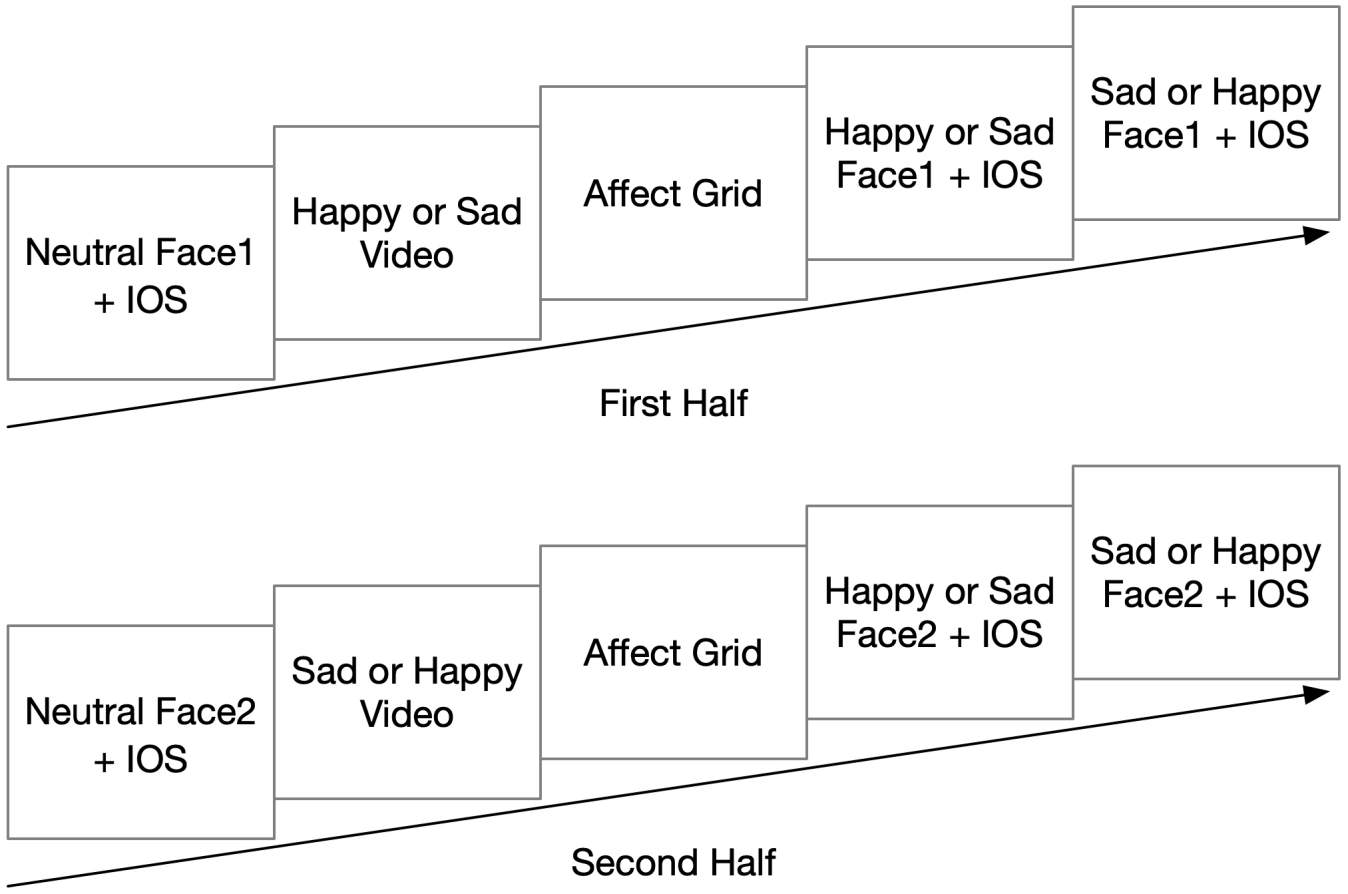
The experimental procedure.

The first half of the experiment ended after two consecutive overlap degree selections. In order to reduce the effect of experience on the subsequent experiments, the participants were asked to solve some simple calculation problems between two parts of the experiment, which lasted for 5 min. The program recorded the number and percentage of correct answers and gave feedback on the correctness during the experiment to attract the participants’ attention to the arithmetic task. The video in the second half conveyed the opposite mood of the first, but the procedure was the same. The whole experimental procedure is shown in Figure 1.

### Data analyses

During the experiment, we thus collected participants’ basic information (age and gender), IOS scores toward neutral faces before watching the videos, AG scores after watching the video, and IOS scores for happy and sad faces after watching the videos. The IOS scores for neutral faces were the baseline of the self-other overlap degree, and the results obtained by subtracting the baseline from the IOS scores toward happy and sad faces after watching the videos represent how self-other overlap changed. Specifically, we reorganized the data and obtained arousal and valence scores after watching the happy/sad videos, which were used to validate the emotional priming effects. Also, IOS changes (IOS scores of neutral faces vs. IOS scores of happy/sad faces) before and after watching the videos were calculated. The reason for choosing IOS changes instead of raw scores as the dependent variable was to consider the individual differences between the subjective judgments of different participants; therefore, the degree of change would better reflect the interaction between watching happy/sad videos and the assessment of self-other integration.

## Results

As a manipulation check, we first analyzed the priming effect of video type on participants’ affective states by means of a univariate ANOVA. The effect of video type was significant for both arousal, *F*(1, 79) = 13.053, *p* = 0.001, η_p_^2^ = 0.142 (*M_happy video_* = 4.65, *SD_happy video_* = 2.147, *M_sad video_* = 3.58, *SD_sad video_* = 1.847), and valence, *F*(1, 79) = 220.394, *p* < 0.001, η_p_^2^ = 0.736 (*M_happy video_* = 7.00, *SD_happy video_* = 2.239, *M_sad video_* = 2.25, *SD_sad video_* = 1.555), which indicates that the videos successfully primed the participants’ affective states as intended.

**Figure 2:**
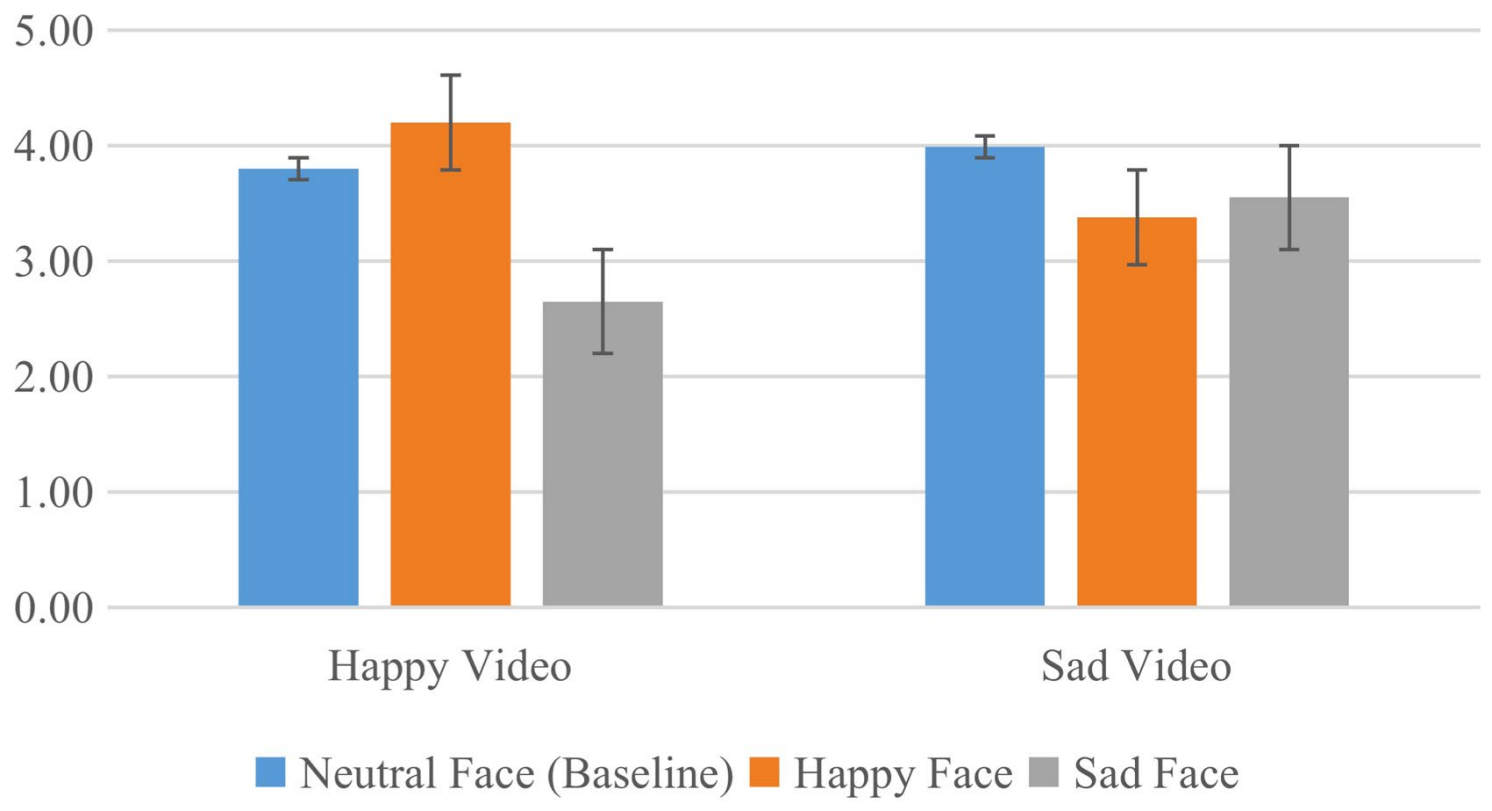
IOS ratings after watching the happy or sad video. Note that the analyses considered the differences between the happy face and sad face conditions to the neutral face baseline.

To test our hypotheses, we then submitted the IOS rating changes (IOS for happy or sad face minus IOS for neutral baseline) to 2 × 2 ANOVA with the factors video type (happy vs. sad video) and facial expression (happy vs. sad face). There was a significant main effect of facial expression, *F*(1, 79) = 20.862, *p* < 0.001, η_p_^2^ = 0.209, due to a drop of IOS for sad faces as compared to happy faces, and a significant interaction, *F*(1, 79) = 25.211, *p* < 0.001, η_p_^2^ = 0.242, indicating that the facial-expression effect was more pronounced after watching the happy video than the sad video. The main effect of video type was far from significant, *F* < 1. Two-tailed paired samples *t-*tests further revealed that, after watching the happy video, IOS scores increased significantly for the happy expression, *t*(79) = 2.489, *p* = 0.015, *d* = 0.278, and decreased significantly for the sad expression, *t*(79) = 4.899, *p* < 0.001, *d* = 0.548, and these two IOS changes differed significantly, *t*(79) = 6.724, *p* < 0.001, *d* = 0.752. In contrast, after watching the sad video, IOS dropped significantly for both happy and sad expressions, *t*(79) = 3.086, *p* = 0.003, *d* = 0.345, and *t*(79) = 2.474, *p* = 0.016, *d* = 0.277, respectively, and these two IOS changes did not differ, *t*(79) = 0.773, *p* = 0.442, *d* = 0.086. Figure 2 provides an overview of the absolute IOS results.

## Discussion

This study aimed to investigate the principles underlying people’s tendency to include others into their self-representation. We specifically focused on the role of affective state and made two predictions. The first was based on TECs feature-overlap principle, which suggests that people would include unfamiliar others more into their self-representation if they are sharing features with them. This predicts that IOS scores should be higher if the expression shown by an unfamiliar face matches (vs. mismatches) the participant’s affective state as induced by the video. The second was based on metacontrol theory, suggesting that people should tend to include others into their self-representation more if they are in a happy mood. We primed happy and sad affective states by means of happy and sad videos, respectively, and measured the degree of self-other inclusion through IOS. Our manipulation checks confirmed that the mood induction worked as expected (indicated by the significant effect of video type for both arousal and valence), suggesting that the videos were effective in inducing happy and sad states in our participants.

The first prediction was confirmed. It amounts to the prediction of an interaction between video type and facial expression, which was indeed significant. The pattern also confirmed our expectations: IOS scores were higher if the video-induced affective state matched the expression shown by the unfamiliar face. This provides evidence for the importance of the feature-overlap principle of TEC for predicting self-other integration. However, the prediction was more successful for positive-mood induction than it was for negative-mood induction. Indeed, the predicted feature-overlap effect was significant for positive-mood induction only, whereas the negative-mood induction resulted in an overall drop and an only numerically higher IOS score for the sad face—far from any conventional significance level. This might have been due to a greater efficiency of the positive-mood induction than of the negative-mood induction, but given the very low valence scores after the sad video (2.25) this seems rather unlikely. Another possibility relates to theoretical suggestions that happy mood facilitates global processing, whereas sad mood boosts local processing (Schmid et al., 2011). According to the affect-as-information hypothesis (Clore and Palmer, 2009), people in a happy mood tend to make judgments based on overall impressions and adopt a more automatic information processing style. In contrast, people in a sad mood are assumed to search for specific information, which in our case might have attracted attention to discriminating rather than shared features. However, this would also fail to explain why the two faces after the sad video received comparable IOS scores (*M_happy face_* = 3.38, *M_sad face_* = 3.55). Such a phenomenon might be explained under the umbrella of metacontrol theory, however (see below).

The second prediction was not confirmed. It amounts to predicting a main effect of video type, which was far from significance. At the same time, however, the metacontrol-inspired prediction accounts for three of the four data points obtained. The prediction worked perfectly well for the sad video, which indeed reduced IOS scores for both kinds of faces. It also worked for the happy faces after the happy video, for which IOS scores increased significantly. The only exception refers to the sad faces after the happy video, for which IOS scores are much lower than for sad faces after the sad video. This suggests that the mechanisms behind our two predictions might not be functionally independent, in the sense that the impact of mood on metacontrol might have repercussions for how mood impacts the processing of feature overlap between self and other. Indeed, this would fit with our observation that the success of the feature-overlap prediction depended on the induced mood.

Taken altogether, this suggests that sharing an affective state with an unfamiliar face increases self-other integration if, and only if, metacontrol is biased toward flexibility, like when being in good mood. It is assumed that flexibility is accompanied by an integrative processing mode that does not strongly distinguish between strictly task-relevant and task-irrelevant information, in contrast to the strong focus on task-relevant information under a persistence bias (Hommel and Colzato, 2017). Given that the feature overlap in terms of affective states between participant and the unfamiliar face was not strictly relevant for IOS score changes, it thus makes sense to assume that the greater flexibility induced by the happy video may facilitate the task-irrelevant information regarding the affective state of the participant and the unfamiliar face. In contrast, the sad video should have induced a more persistent metacontrol state (or at least failed to induce a more flexible state), which, in turn, would lead to the exclusion of information about the affective states of participant and face. As a result, the respective feature overlap between self and other was not considered and, thus, had no impact on the IOS scores. According to this scenario, the affective state of individuals does have an impact on both feature overlap and metacontrol, with possible consequences for the perceived self-other overlap. However, the processing of the information about feature overlap is not independent from the current metacontrol state, so that not strictly task-relevant information is less likely to be considered under negative mood.

As an aside, we found that participants’ baseline level of IOS ratings before watching the video was close to moderate (*M* = 3.894, *SD* = 1.654), indicating a certain overlap between the participants and the synthetic photos they saw. Such a result may be due to the fact that the photos we used in the experiment were synthesized from real photos (each synthesized face was composed of eight different photos with the same gender). Although different individuals look very different, there may be some similarities between these differences, which could be one of the bases for the ability of human beings to empathize with others. However, this remains a speculation that calls for future research.

## Conclusion

The present study provides new insights into understanding the role of affective states in self-other representation. Our findings suggest that positive mood promotes the processing of features that might lead to self-other overlap, which, in turn, facilitates including the other into one’s self-representation. Negative mood, in turn, seems to foster self-other distinction and exclusion of others.

## Data availability statement

The raw data of this study are archived in the Open Science Framework and can be accessed through https://osf.io/9324r/.

## Ethics statement

Ethical approval for this study was obtained from the Psychology Research Ethics Committee of Hangzhou Dianzi University, and written informed consent was obtained from all participants.

## Author contributions

JZ and BH: design of the study. JZ: experimentation, data collection, analysis, and first draft of the manuscript. BH: critical suggestions and revision of the manuscript. All authors contributed to the article and approved the submitted version.

## Funding

This research was supported by the Zhijiang Youth Project of Zhejiang Province Philosophy and Social Science Planning (22ZJQN42YB), National Social Science Fund of China (20FZXB017), as well as China Scholarship Council (CSC).

## Conflict of interest

The authors declare that the research was conducted in the absence of any commercial or financial relationships that could be construed as a potential conflict of interest.

## Publisher’s note

All claims expressed in this article are solely those of the authors and do not necessarily represent those of their affiliated organizations, or those of the publisher, the editors and the reviewers. Any product that may be evaluated in this article, or claim that may be made by its manufacturer, is not guaranteed or endorsed by the publisher.
